# Resolution of acute motor axonal neuropathy in a patient after treatment with efgartigimod: A case report

**DOI:** 10.1097/MD.0000000000040700

**Published:** 2024-12-06

**Authors:** Jinli Zhou, Weifei Yu, Siqi Ding, Chanhong Shi, Hui Liang

**Affiliations:** a Department of Neurology, Yiwu Hospital Affiliated to Wenzhou Medical University, Yiwu Central Hospital, Yiwu, China; b Department of Neurology, The First Affiliated Hospital, Zhejiang University, Medical School, Hangzhou, China.

**Keywords:** acute motor axonal neuropathy, efgartigimod, FcRn inhibitor, IgG

## Abstract

**Background:**

Guillain-Barré syndrome (GBS) is an acute autoimmune neuropathy characterized by progressive muscle weakness, often caused by immunoglobulin G (IgG) autoantibodies. There are several subtypes of GBS, of which acute motor axonal neuropathy (AMAN) is one of the most severe subtypes associated with axonal damage. It is well known that the current clinical standard of treatment is intravenous immunoglobulin (IVIg) and plasma exchange (PLEX), but some patients often show limited response or experience persistent disability. Efgartigimod, an Fc fragment of human IgG antibody, provides a way to target and reduce pathogenic IgG antibodies as a natural ligand Fc receptor (FcRn). The purpose of this study was to observe the therapeutic effect of efgartigimod on axonal GBS, which is expected to be a potential therapeutic method for GBS and AMAN.

**Methods:**

We present a case of a 58-year-old man diagnosed with AMAN, presenting with ascending symmetrical limb weakness, flaccid paralysis, and multiple cranial nerve palsies. Electromyography confirmed the axonal subtype of GBS. Despite receiving IVIg and PLEX, the patient showed suboptimal recovery. Subsequently, he was treated with efgartigimod at a dose of 10 mg/kg weekly for 4 weeks, demonstrating significant improvement in both clinical symptoms and electromyographic findings, with good tolerability.

**Result:**

This case highlights the potential efficacy and safety of a 4-dose efgart-igimod regimen in AMAN, particularly for patients with inadequate response to conventional therapies. By targeting FcRn and promoting IgG degradation, efgartigimod offers a novel mechanism to modulate the aberrant immune response underlying AMAN.

**Conclusion:**

Efgartigimod at a dose of 10 mg/kg weekly for 4 weeks demonstrated promising results in this case of AMAN. While further research is warranted, our findings suggest that efgartigimod may represent a valuable addition to the therapeutic armamentarium for AMAN and potentially other autoimmune neurological conditions. Well-designed clinical trials are crucial to confirm these findings and establish optimal treatment protocols for efgartigimod in AMAN.

## 1. Introduction

Acute motor axonal neuropathy (AMAN), an axonal variant of Guillain-Barré syndrome (GBS), was first described by Feasby et al in 1986. AMAN typically manifests as rapidly progressive limb weakness, which can lead to respiratory failure and, in some cases, cranial nerve dysfunction and autonomic disturbances.^[1]^ GBS is an autoimmune disorder mediated by immunoglobulin G (IgG) autoantibodies, often triggered by molecular mimicry following an infection.^[2–4]^ The neonatal Fc receptor (FcRn) plays a crucial role in regulating IgG homeostasis by protecting IgG from degradation and extending its half-life.^[5]^ Efgartigimod is the first neonatal Fc receptor (FcRn) antagonist approved for the treatment of generalized myasthenia gravis. Previous research has demonstrated the safety and efficacy of efgartigimod in treating GBS, using a 2-dose regimen.^[6]^ Herein, we report on 1 AMAN patient treated with efgartigimod at a dose of 10 mg/kg as 4 infusions (1 infusion per week), a regimen that has never been reported before.

## 2. Case presentation

A 58-year-old man was admitted the hospital who presented with acute lower limb weakness of 3 days duration, following a recent febrile illness. He subsequently developed progressive weakness in the arms and face, along with slurred speech and difficulty swallowing. Initial neurological examination revealed several cranial nerve abnormalities, including bilateral facial weakness with a mask-like appearance, dysarthria due to palatal weakness, and dysphagia suggesting bulbar involvement. Deep tendon reflexes were diminished (+1) throughout. Muscle strength, graded using the Medical Research Council Scale, was 0/0/0/2 proximally to distally in the upper limbs and 0/0/0/1 in the lower limbs. Electrophysiological studies (electromyography [EMG] nerve conduction studies) showed partial motor nerve conduction block without sensory involvement (Table 1), and cerebrospinal fluid analysis demonstrated albuminocytological dissociation, confirming the diagnosis of AMAN, even though serum anti-ganglioside antibodies (GM1, GM2, GM3, GM4, GD1a, GD1b, GD2, GD3, GT1a, GT1b, GQ1b, and sulfatide) were negative.

**Table 1:** The changes of electrophysiological examination.

			Time of onset	After 4 doses	2-month follow-up
Median	Left	CMAP (mV)	2.2	5.9	7.2
		MCV (m/s)	50.3	52.7	54.2
		SCV (m/s)	52.0	50.1	53.8
	Right	CMAP (mV)	2.1	6.4	8.4
		MCV (m/s)	48.0	49.1	51.0
		SCV (m/s)	52.5	53.5	54.0
Ulnar	Left	CMAP (mV)	2.1	4.2	6.9
		MCV (m/s)	51.2	53.1	55.7
		SCV (m/s)	51.3	53.0	53.8
	Right	CMAP (mV)	2.4	3.9	7.8
		MCV (m/s)	51.3	51.5	52.5
		SCV (m/s)	52.3	53.1	53.8
Common peroneal	Left	CMAP (mV)	2.3	4.4	6.2
		MCV (m/s)	46.2	47.3	48.1
		SCV (m/s)	45.1	46.5	46.6
	Right	CMAP (mV)	2.7	5.1	7.5
		MCV (m/s)	47.1	47.3	48.5
		SCV (m/s)	46.3	46.8	49.4

CMAP = compound motor active potential, MCV = motor conduction velocity, SCV = sensory nerve conduction velocity.

The patient received intravenous immunoglobulin (IVIg) at 400 mg/kg for 5 days, but subsequently developed respiratory failure requiring mechanical ventilation and plasma exchange (PLEX) once a day for 5 days in the intensive care unit. Five days later, he was weaned off ventilation and transferred to the neurology ward with persistent limb weakness(GBS disability score of 4). Treatment with 10 mg/kg of efgartigimod was initiated >1 month after onset and 2 weeks after IVIg and PLEX. Following the first infusion, the patient regained the ability to sip water. After the second dose, he could raise his arms and bend his legs. By the fourth dose, his upper limb strength had improved to grade 5, and lower limb strength to grade 4 (GBS disability score of 3). Follow-up EMG nerve conduction studies at 3 weeks showed significant improvement in nerve conduction velocity (Table 1).

At a 2-month follow-up assessment, the patient demonstrated significant functional and EMG improvement (Table 1), with GBS disability score of 2. This score indicates slight disability but the ability to walk without assistance. He had mild residual weakness persisting in his upper legs.

## 3. Discussion

AMAN, characterized by axonal damage, represents a more severe subtype of GBS. GBS itself is an immune-mediated neuropathy triggered by various factors, including infections, vaccines, and toxins.^[7,8]^ Early initiation of immunotherapy is crucial to prevent irreversible nerve damage and improve clinical outcomes. IVIg and PLEX are the mainstay of GBS treatment. IVIg is often preferred due to its ease of administration and favorable side effect profile.^[9–12]^ It is typically administered at a dose of 0.4 g/kg for 5 days. While the exact mechanisms are complex, IVIg is thought to neutralize pathogenic antibodies, inhibition of Fc-mediated macrophage activation and complement activation, modulation of T-cell and B-cell functions. PLEX, involving the removal and replacement of a patient’s plasma, is another effective option, particularly in severe cases. Its mechanisms likely involve the removal of circulating autoantibodies and inflammatory mediators.

IVIG and PLEX, which were efficacious immunotherapies for patients with GBS, should be initiated as early as possible before irreversible peripheral nerve injury occurs.^[9–11]^ IVIG (0.4 g/kg for 5 days) is the first-option therapy, as it is more convenient to administer and has fewer side-effects.^[10,12]^ The pharmacological mechanism of IVIG may be associated with neutralization of antibodies, inhibition of Fc-mediated macrophage activation and complement activation, modulation of T-cell and B-cell functions. PLEX (typically involving 5 sessions of 200–250 mL/kg) is another effective treatment that significantly improves clinical outcomes for GBS patients in the early stages.^[13]^ Although the exact mechanisms of action remain unclear, PLEX is thought to exert its therapeutic effects by removing circulating autoantibodies, immune complexes, complement factors, and pro-inflammatory cytokines.^[13]^ IVIg and PLEX are not always available, especially given the growing global shortage of blood products. In addition, it has been documented IVIg takes 2 weeks to become effective.^[14]^ And patients treated with IVIg commonly experienced side effects (headache, tachycardia, nausea, vomiting, etc), and 2% to 6% of patients had serious side effects (thrombosis, aseptic meningitis, renal insuffciency, neutropenia, etc).^[15]^ PLEX can show clinical effects within the first week of treatment,^[14]^ but it may also remove immune complexes and cytokines and can induce arrhythmias and hypotension.^[16]^ Furthermore, despite the application of conventional immunotherapeutic approaches, around 3% to 7% of patients die, and 20% remain unable to walk after 6 months.^[17]^ Therefore, the development of targeted immunotherapies with novel mechanisms of action represents a crucial advancement in the quest for improved GBS treatment strategies.

Immunoglobulin G (IgG)is the most common antibody and the main efficacious molecule of humoral immune response.^[18]^ Several types of IgG Fc receptors, including FcRn, are found on the surface of immune cells and play a role in mediating immune responses. The FcRn is an MHC class I-like molecule that recycles IgG, extending its half-life by about 4 times that of other immunoglobulins that are not recycled by FcRn (e.g., IgM or immunoglobulin A).^[19]^ Following cellular uptake, the Fc region of an IgG antibody binds 2 FcRn receptors under acidic conditions in the endosome.^[20]^ IgGs bound to FcRn are rescued from lysosomal degradation and released at physiological pH outside the cell.^[5,21–23]^ The ability to inhibit FcRn and subsequently reduce autoantibody levels provides a novel and targeted approach for treating autoimmune diseases, offering hope for improved therapeutic outcomes.^[24]^ Efgartigimod is a specifically designed molecule that binds to FcRn with high affinity, competing with natural IgG and inhibiting its recycling.^[21,22]^ leading to a reduction in overall IgG levels, offering an effective and well-tolerated treatment option for autoimmune diseases.^[25]^

A recent clinical study demonstrated the effectiveness of a 2-dose efgartigimod regimen (10 mg/kg) in treating GBS patients.^[6]^ Since our patient had minimal improvement with IVIg and PLEX, we administered efgartigimod at 10 mg/kg weekly for 4 weeks and the patient was responded well to the treatment.The GBS disability score of the patient was 4 at admission, and the GBS disability score remained 4 after administration of IVIg and PLEX. Two months after administration of efgartigimod, the GBS disability score decreased by 2 compared with before. At the same time, the IgG level of the patient was 15.2 g/L at admission and 8.4 g/L at the end of t months after the use of efgartigimod, indicating a decrease. Our study result showed that the patient’s clinical symptoms, electromyogram and IgG level improved significantly after treatment of efgartigimod and albumin was not reduced by post-treatment monitoring. Efgartigimod is well tolerated in our patient. Patient experienced muscle soreness after treatment. In fact, myalgia is common in GBS patients and might be not associated with efgartigimod.

### 3.1. Limitations

Encouragingly, patient achieved favorable outcomes, bolstering confidence in using efgartigimod for treating AMAN and other IgG-mediated immune-related disorders. However, this article is only 1 case report, and clinical studies involving more centers and more cases are needed to confirm its effectiveness.

## 4. Conclusion

This case report suggests the potential efficacy and safety of a 4-dose efgartigimod regimen (10 mg/kg weekly) for AMAN patients with suboptimal response to IVIg or PLEX. The positive outcomes observed in this case suggest that efgartigimod may hold promise for treating other autoimmune neurological conditions with similar underlying mechanisms.

## Author contributions

**Investigation:** Siqi Ding.

**Resources:** Weifei Yu.

**Supervision:** Chanhong Shi.

**Writing – original draft:** Jinli Zhou.

**Writing – review & editing:** Hui Liang.
